# Developmental Origin of the Cardiac Conduction System: Insight from Lineage Tracing

**DOI:** 10.1007/s00246-018-1906-8

**Published:** 2018-05-17

**Authors:** Rajiv A. Mohan, Bastiaan J. Boukens, Vincent M. Christoffels

**Affiliations:** Department of Medical Biology, University of Amsterdam, 1105 AZ Amsterdam, The Netherlands

**Keywords:** Lineage tracing, Cardiac conduction system, Genetic inducible fate map, Cardiac development

## Abstract

The components of the cardiac conduction system (CCS) generate and propagate the electrical impulse that initiates cardiac contraction. These interconnected components share properties, such as automaticity, that set them apart from the working myocardium of the atria and ventricles. A variety of tools and approaches have been used to define the CCS lineages. These include genetic labeling of cells expressing lineage markers and fate mapping of dye labeled cells, which we will discuss in this review. We conclude that there is not a single CCS lineage, but instead early cell fate decisions segregate the lineages of the CCS components while they remain interconnected. The latter is relevant for development of therapies for conduction system disease that focus on reprogramming cardiomyocytes or instruction of pluripotent stem cells.

## Introduction

The cardiac conduction system (CCS) coordinates the rhythmic contractions of the atria and the ventricles by tightly controlling the generation and propagation of the electrical impulse. Failure to correctly pattern and develop the CCS components leads to their dysfunction and may result in arrhythmias such as atrioventricular block or sick sinus syndrome [1]. Gaining insight into the developmental processes and cell populations involved in the formation of the CCS is important to understand the etiology of these arrhythmias.

The omnipotent zygote grows into a mature multicellular organism consisting of hundreds of different specialized cell types. This occurs through cell fate decisions made during development that restrict their possible fates. A lineage of a mature and specialized cell type encompasses the developmental history (or ancestry) traced back to the zygote. More often a lineage is traced back to a progenitor in which a lineage decision was made. Such a decision point often represents a branching point leading to multiple lineages. Knowledge of the consecutive cell fate decisions made during the development of the CCS provides insight into the underlying molecular mechanisms.

A variety of experimental approaches have been used to interrogate the lineage decisions leading to the formation of the CCS, which have been interpreted in different ways. In this review, we briefly summarize the composition and function of the CCS components and the common developmental history of the cells comprising them. The main part of this review covers the available data on the CCS lineages and techniques used to trace the CCS lineages. Finally, we present a model for the CCS lineages. Our literature research indicates that early in embryonic development multiple CCS lineages are formed leading to sharp boundaries between the CCS components they will form.

### The Cardiac Conduction System Components—Location and Function

**Fig. 1:**
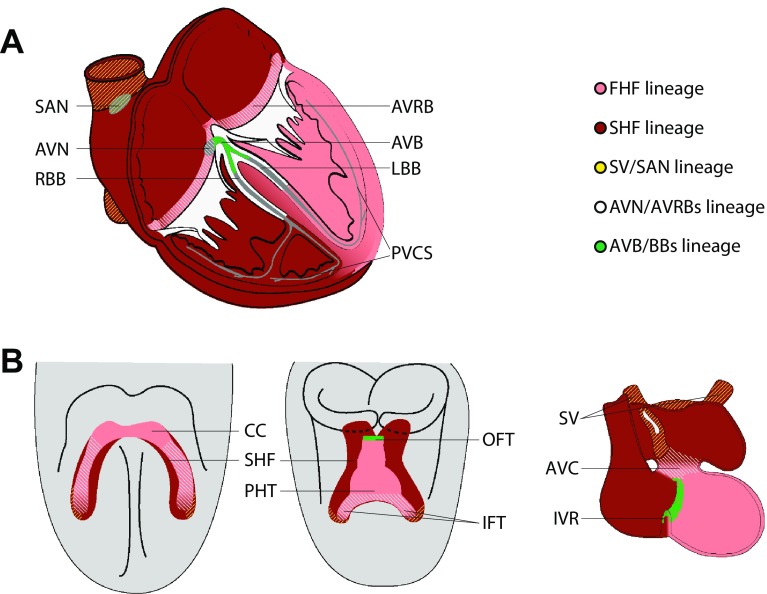
Developmental origin of the cardiac conduction system components. **a** The definitive cardiac conduction system (CCS, gray) consists of the sinoatrial node (SAN), the atrioventricular node (AVN) and ring bundles (AVRBs), the atrioventricular bundle (AVB), left and right bundle branches (LBB and RBB, respectively) and the peripheral ventricular conduction system (PVCS). The first and second heart field (FHF and SHF, respectively) contributions to the adult myocardium are visualized in pink (FHF-derived) and dark red (SHF-derived). **b** Mesodermal cells in the FHF differentiate and form the cardiac crescent (CC) at E7.5. Fusion of the cardiac crescent (E8) gives rise to the primary heart tube (PHT) and subsequently forms the left ventricle and part of the atrioventricular canal (AVC). Cardiogenic mesodermal cells in the SHF are continuously added to both poles of the heart tube and form the other myocardial components of the heart. The locations of the progenitor cells of the CCS components are depicted by yellow lines for the SV/SAN, blue lines for AVN/AVRBs and green dots for AVB/BBs. *IFT* inflow tract, *IVR* interventricular ring, *OFT* outflow tract, *SV* sinus venosus. Reproduced with permission from [3]

In the adult heart, the initial electrical impulse is generated within the CCS in a regular rhythm and propagates through the atrial and ventricular myocardium, triggering calcium release that results in contraction (Fig. 1a). The sinoatrial node (SAN) is the dominant pacemaker of the heart generating the electrical impulse and is located at the border of the superior caval vein and the right atrium. The impulse spreads through the atria and reaches the atrioventricular node (AVN) and ring bundles (AVN and AVRBs, respectively). The AVN forms the only myocardial (i.e., electrical) connection with the ventricular conduction system (VCS) and is located dorsally at the base of the interatrial septum. The AVN propagates the impulse very slowly. In contrast, the VCS consisting of the (1) atrioventricular bundle (AVB) within the crest of the interventricular septum, (2) left and right bundle branches (LBB and RBB), and (3) the left and right peripheral ventricular conduction system (PVCS) or Purkinje fiber network, all of which rapidly conduct the impulse. The PVCS forms an complex cellular network that, in human and rodents, lies just below the endocardium, and in other mammals and birds branches deeper into the compact ventricular wall. Finally, the electrical impulse reaches the ventricular working cardiomyocytes, thereby initiating their depolarization followed by calcium-induced calcium release and contraction, resulting in pumping blood into the aorta and pulmonary artery [2].

Cardiomyocytes from the CCS share important features related to their specialized function such as spontaneous depolarization rate, degree of intercellular coupling and action potential morphology. These features, however, differ significantly between CCS components, suggesting that there are multiple distinguishable CCS lineages. For example, the SAN is the dominant pacemaker because its spontaneous depolarization rate is higher than that of the other CCS components. Moreover, both the SAN and AVN conduct the impulse slowly, whereas the VCS conducts it rapidly [4–6]. These particular features of CCS components are important for their proper function and result from different transcriptional programs that are established through lineage decisions during embryogenesis.

### Origin of the Heart Components—General Concept

The cardiogenic mesoderm forms just after gastrulation at the cranio-lateral side of the forming embryo. The cranial parts of the two bilateral populations fuse at the midline giving rise to the cardiac crescent. Multiple developmental processes, including folding of the embryo, foregut formation and fusion of the cardiac crescent in a craniocaudal direction result in the formation of the primary heart tube. In chicken embryos, microparticle labeling of the initial primary heart tube revealed that it is fated to form the LV [7]. Based on *Hcn4*^+^ and *Tbx2*^+^ lineage tracing (see below), the mouse cardiac crescent is thought to form the LV and part of the AVC [8, 9]. At the primary heart tube stage, growth of the heart does not occur by proliferating cardiomyocytes [10]. At this stage, the heart grows by addition (or accretion) of surrounding cardiogenic mesoderm to the venous and arterial pole of the heart tube, giving rise to the remainder of the definitive heart. This was demonstrated by a multitude of approaches including dye labeling, transgenic labeling using the long half-life of β-galactosidase, viral transduction, in vivo ablation and Cre-mediated lineage tracing experiments [10–14]. This progenitor pool is referred to as the second heart field (SHF). At the inflow of the heart, the primary heart tube’s inflow tract becomes the major part of the AVC. Newly added inflow tract cells are fated to form the atria. At last, the cells of the definitive SV are added. The first cells added from the SHF to the arterial pole form the outflow tract of the primary heart tube which subsequently becomes the RV. Cells added thereafter form the fetal outflow tract and subsequently the RV outflow tract. Finally, the last added cells give rise to the intra pericardial portion of the aorta and pulmonary trunk. In summary, the FHF forms the initial primary heart tube that subsequently forms the definitive LV and part of the AVC. The SHF in the dorsal pericardial wall forms all other structures within the heart.

In the developing heart, the SAN forms within the SV myocardium at the border with the RA. The AVN forms within the inferior AVC, and may originate from the FHF-derived IFT of the primary heart tube. The location of the AVB and BBs on the crest of the IVS, in-between the LV (FHF-derived) and RV (SHF-derived) suggests that the AVB is derived from progenitors at the border between the FHF and anterior SHF, the LBB from FHF, and RBB from SHF progenitors. Accordingly, the left ventricular PVCS forms from cells originating from the primary heart tube derived from the FHF. The right ventricular PVCS is formed within the RV compartment, a SHF-derived structure that is added to the primary heart tube at the arterial pole (Fig. 1b).

### The Sinoatrial Node is Formed by Early Cell Fate-Restricted Mesodermal Cells

**Fig. 2:**
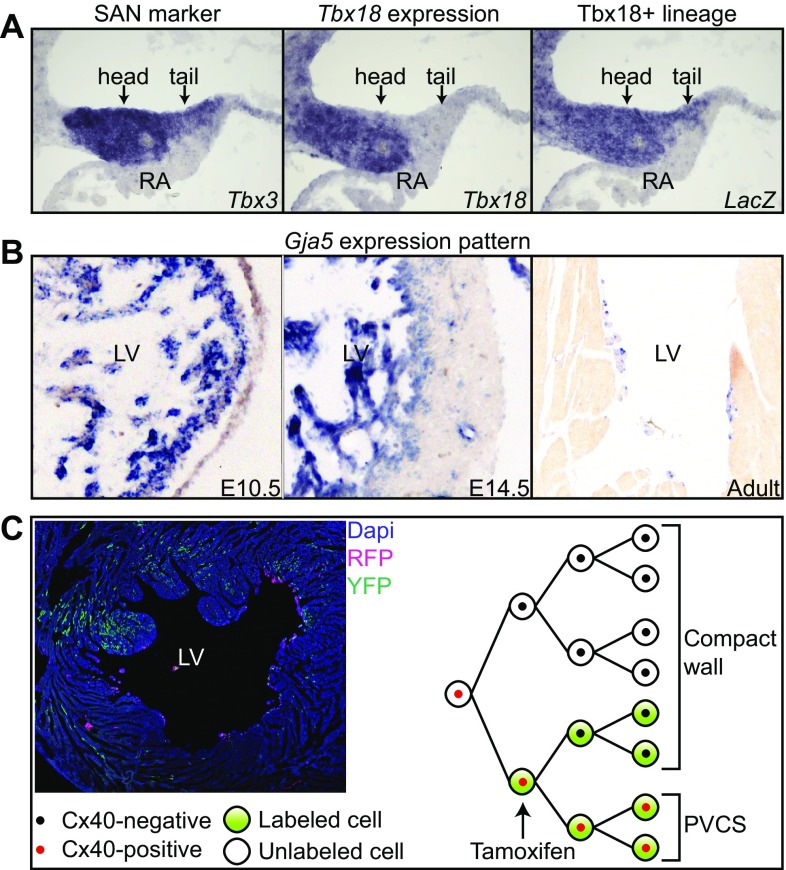
Lineage tracing identifies CCS progenitors and reveals its mode of development. **a** The definitive SAN includes a ‘head’ and ‘tail’ part, both expressing *Tbx3. Tbx18* is specifically expressed in the ‘head’ and not in the ‘tail.’ However, using Tbx18-Cre;R26R^LacZ/LacZ^ mice, the *Tbx18*^+^ progenitors were found to contribute to both parts of the SAN. **b** Spatio-temporal expression pattern of *Gja5* at several stages. At E10.5 *Gja5* is expressed transmurally in the embryonic left ventricular myocardium. At E14.5, expression is downregulated in the compact myocardium of the left ventricle and maintained within the trabeculae. At the adult stage, *Gja5* is only expressed in the PVCS of the left ventricle. **c** Labeling E10.5 embryonic *Gja5*^+^ cardiomyocytes using Tamoxifen in an inducible Cre model (*Gja5-CreER*^*T2*^*-IRESmRFP;R26R-YFP*) shows a contribution to the formed PVCS and ventricular working myocardium demonstrating a common origin from *Gja5*^+^ cells and downregulation of *Gja5* in the compact myocardium. *Tbx18* lineage tracing is adapted from [17], *Gja5* expression pattern from [23], and the *Gja5* lineage tracing are unpublished results from [24]

The SAN, together with the left, common and right sinus horns and the venous side of the venous valves, form the definitive SV. During mouse development, the SAN primordium can be discriminated morphologically around E10 within the embryonic SV directly adjacent to the embryonic right atrial wall [15]. The T-box transcription factor Tbx18 is selectively expressed in the SV/SAN as opposed to *Nkx2-5* that is expressed within the FHF, SHF, and all other embryonic heart components [16, 17]. Cre-based genetic lineage analysis using *Tbx18*-driven Cre and *Nkx2-5*-driven Cre demonstrated that the SV and SAN are derived from *Tbx18*^+^ *Nkx2-5*^−^ precursors (Fig. 2a). As a consequence, a sharp lineage boundary was present between the SV/SAN and the *Tbx18*^−^ *Nkx2-5*^+^ *Nppa*^+^ atrial myocardium [16–18]. Complementary, labeling of embryonic atrial cardiomyocytes from E10.5 onwards using an *Nppa*-driven Cre did not result in labeled descendants within the SV/SAN [19]. These three genetic labeling experiments clearly indicated that SAN cardiomyocytes do not originate from *Nppa*^+^ *Nkx2-5*^+^ atrial cardiomyocytes, but from *Tbx18*-expressing SV progenitors [16–19]. In line, Mommersteeg et al. showed that *Tbx18*^+^ mesodermal cells differentiated into *Nkx2-5*^−^ cardiomyocytes after culturing in vitro. Moreover, these explants had a higher beating frequency compared to explants from embryonic ventricular cardiomyocytes [17, 20]. An alternative approach in which the *Tbx18*^+^ pericardial wall was labeled using a dye further indicated that the *Tbx18*^+^ mesoderm includes the SV progenitor population [20]. The transcription factor Short stature homeobox 2 (Shox2) becomes expressed in the SV at E9.5, and remains expressed in the SAN [21]. *Shox2*-driven Cre specifically labeled the SV/SAN. Both Tbx18 and Shox2 specify the SV/SAN lineage and are important for the formation of the SAN [17, 22].

Dye labeling of small regions of cultured mouse embryos at the 4- and 6-somite stage revealed that cranially located posterior SHF cells contribute to the AVC and atria, whereas cells more caudally contribute to the SV [25]. Another study using dye labeling in chicken embryos demonstrated that the SAN progenitors in the early embryo are located in a region posterior of the *Nkx2-5*^+^ and *Isl1*^+^ lateral plate mesoderm. The relative position of the mesodermal SAN progenitors may be similar to the *Tbx18*^+^ *Nkx2-5*^−^ progenitor mesoderm in the mouse, although the latter were mapped at a later embryonic stage. Interestingly, specification of the SAN progenitors was found to be established when they are still part of the cardiogenic mesoderm, revealing an early segregation of the SV/SAN lineage [26].

The *Nkx2-5-Ires-Cre* lineage tracing study indicated a sharp boundary between *Nkx2-5*^+^-derived atrial cardiomyocytes and *Nkx2-5*^−^-derived SV/SAN cardiomyocytes [18, 27]. Using different genetic tools, the SV progenitor population was indicated to express *Nkx2-5* [8]. Previous work focused on the SV/SAN cardiac progenitors demonstrated *Nkx2-5* and *Isl1* expression for a brief period around E7.0. The expression of both genes is downregulated before *Tbx18* becomes expressed [20, 28, 29]. The brief period of *Nkx2-5* expression as well as the use of other reporters could explain the absence of SAN labeling in the *Nkx2-5-Ires-Cre* experiment [20, 27]. Altogether, *Tbx18*^+^ mesodermal cells briefly express *Nkx2-5* and *Isl1* before differentiating into cardiomyocytes that form the SV/SAN. To study the *Isl1*^+^ progenitor population an *Isl1-Cre* and *Isl1-MerCreMer* have been used in combination with different reporters [8, 11, 30, 31]. The first *Isl1*^+^ lineage tracing did not specifically mention labeling of the SAN. The IFT of the E8.5 heart tube, however, contained labeled cells [11]. Yet another *Isl1-Cre* fate map experiment showed near complete labeling of SAN cardiomyocytes [8]. Nonetheless, a contribution from the *Isl1*^+^ progenitor population to the SAN remained obscure because *Isl1* is still expressed in the mature SAN [32, 33]. The latter can be solved by temporal control of labeling using an inducible Cre. For instance, specific labeling at E6.0 using *Isl1*-*MerCreMer* resulted in labeling of the SV. Sparse labeling of the SAN (identified based on morphology, not specific markers) was observed after labeling at E9.0 and fate analysis at E11.0 [30]. Within the caudal SHF, two expression domains are present, a *Tbx18*^+^ and an *Isl1*^+^ domain. These expression domains do not overlap except for one region where shortly thereafter the embryonic SAN cardiomyocytes (*Tbx18*^+^, *Isl1*^+^, *Tbx3*^+^, and *Hcn4*^+^) can be discriminated [20].

In conclusion, accretion of SHF progenitors at the venous pole gives rise to the atria and SV that includes the SAN. Lineage tracing revealed that these two lineages originate from progenitor pools that segregate early in development within the caudal SHF.

### The AVN and AVRBs Originate from the Atrioventricular Canal

The developmental origin of the AVN, AVRBs, AVB, and BBs was inferred from spatio-temporal expression patterns of key markers during heart development. G1N2 is a neural tissue antigen expressed in the AVN, AVB, and BBs of the formed heart. At earlier development stages, the interventricular ring, which includes the crest of the forming interventricular septum and the right-sided hemisphere of the AVC, expresses G1N2. The G1N2^+^ cardiomyocytes in the crest of the interventricular septum extend at both sides into the trabecular ventricular walls. This expression pattern co-localized with the expected positions of the progenitor populations of the AVN, AVB, and BBs [34]. An almost similar expression pattern was observed for the T-box transcription factor *Tbx3*, except that its expression pattern includes the entire AVC (and SAN) [35]. Finally, a transgenic mouse carrying an enhancer of chicken *Gata6* (*cGata6*) coupled to a reporter gene showed activity specifically in the AVN and AVB cardiomyocytes at E14.5. The *cGata6* enhancer was also active in the AVC at E9.5, and was seen to be activated around E7.5 in the posterior region of the cardiac crescent [36]. Therefore, the AVN progenitors seem to originate from the AVC which can be traced back to the posterior part of the cardiac crescent.

Formation and patterning of the AVC involves repression of the working myocardial gene program by Tbx3 and the closely related T-box transcription factor Tbx2 [9, 35, 37]. Within the AVC, myocardial patterning is abrogated when at least three functional alleles of *Tbx2*/*Tbx3* are missing, revealing functional redundancy of these factors in patterning of the AVC [37]. *Tbx2* expression is observed within the caudal extensions of the cardiac crescent. The AVN, AVRBs, and base of the LV derive from this *Tbx2*^+^ population [9]. In contrast, the VCS does not originate from the *Tbx2*^+^ population suggesting that the VCS, although forming a myocardial continuity with the AVN, segregates before *Tbx2* becomes expressed in the cardiac crescent. Early segregation of the AVN and AVB was also indicated by an *nLaacZ-*based retrospective clonal analysis [38]. Fate analysis of the *Tbx2*^+^ lineage at E8.5 and E9.5 showed respectively only labeled cells in the *Tbx2*^+^ IFT and *Tbx2*^+^ AVC [9]. This suggests that the *Tbx2*^+^ cardiogenic mesoderm cells form the embryonic IFT, subsequently the AVC and ultimately the AVN and AVRBs. Interestingly, *Tbx2* expression in the cardiac crescent corresponds with the site of *cGata6* enhancer activity at that stage [9, 36]. Lineage analysis using *cGata6*-Cre showed labeling of the AVN and AVB. Moreover, the *cGata6* lineage, like the *Tbx2*^+^ lineage, contributes to the LV where the *cGata6* enhancer itself is not active. Both the *Tbx2*^+^ and *cGata6*^+^ lineage tracing experiment have the limitation that the AVN in the formed heart maintains expression of both markers at the moment of fate analysis, thus precluding the conclusion that the *Cre*^+^ cells earlier in development represent the AVN progenitors [9, 36]. However, fate analysis at E8.5 and E9.5 of the *Tbx2*^+^ lineage showed labeled IFT and AVC cells, and therefore it is reasonable to assume that the descendants of these cells are also labeled in the fate map analysis in the formed heart [9]. Alternatively, but in line, Tbx2 is required for the AVC myocardial phenotype and most probably involved in the specification of the AVC/AVN lineage [37]. In conclusion, the posterior, or caudal, part of the cardiac crescent forms the AVC which in turn gives rise to the AVN and AVRBs.

Although the *Tbx2* and *cGata6* enhancer lineage analyses point to the progenitor population of the AVN and AVRBs it does not exclude the possibility that other cell populations contribute as well. Other Cre-based genetic lineage experiments exclude contributions from particular lineages to the AVN. At E8.5, *Mef2c-AHF-Cre* is expressed in the anterior part of the SHF. Fate analysis of these *Cre*^+^ progenitor cells showed contributions to the RV, outflow tract and interventricular septum including the AVB [39]. However, no contribution was observed from *Mef2c-AHF-Cre*^+^ cells to the AVN at any developmental stage [9]. In fact, a sharp boundary was present between the *Tbx2*^+^-derived AVC/AVN and the *Mef2c-AHF-Cre*^+^-derived *Gja5*^+^ AVB, including the *Gja5*^+^ lower nodal cells. *Tbx18* is expressed in the epicardium and SV. *Tbx18*^+^ lineage analysis excluded a contribution of these *Tbx18*^+^ tissues to the AVN cardiomyocytes. The connective tissue surrounding the AVN cardiomyocytes, however, was labeled and is a derivative of the *Tbx18*^+^ epicardium [40]. In another study, a *Wnt1*^+^ neural crest lineage tracing experiment (*Wnt1-Cre*) suggested that the neural crest contributes cells to the VCS [41]. However, a more careful examination of the fate map showed that *Wnt1*^+^ cells do not contribute to the *Gja5*^+^ cardiomyocytes of the VCS. The majority of the descendants of the neural crest cells around the *Gja5*^−^ *Tbx3*^+^ AVN cardiomyocytes do not express *Tbx3*, suggesting that neural crest cells do not differentiate towards a AVN cardiomyocyte phenotype [38]. A recently published paper using *in ovo* dye labeling showed that SV cells contribute to the posterior region of the AVC. Initially, the SV and AVC are physically separated; however, later in development they fuse and form a continuity, and part of the SV becomes incorporated into the posterior AVC [42]. This contradicts the *Tbx18* lineage tracing in which such a contribution to the AVN or AVRB cardiomyocytes was not observed [20, 40]. This discrepancy can be explained by an incomplete *Tbx18*^+^ fate map, mouse-chicken differences, or by incorrect identification of the AVC in the dye labeling experiment, as the AVC was identified based on morphology but not on genetic marker expression [42]. Altogether, it is likely that the AVN forms primarily from the *Tbx2*^+^ *cGata6*^+^ AVC that in turn is derived from posteriorly located cardiogenic mesoderm.

### Ventricular Conduction System: Segregation of Embryonic Trabeculae, Interventricular Ring, and Compact Myocardium

The adult VCS, as opposed to the other ventricular cardiomyocytes and the AVN, expresses *Gja5* (encoding connexin subunit Cx40) that allows for fast propagation of the electrical impulse (Fig. 2b). *Gja5* is expressed at higher levels at the left than at the right side of the interventricular septum. The LBB consists of several branches running towards the apex, while the RBB consists of only one branch. The left and right ventricular PVCS are also different in morphology. The left ventricular PVCS is present on the IVS. In contrast, the right ventricular PVCS extends towards the free wall of the RV [43].

The spatio-temporal expression patterns of *Gja5, Nppa* and *CCS-LacZ* provided a prediction of the process of PVCS specification [44, 45]. In mouse (E9-9.5) and chicken chamber-forming hearts, the expression of *Gja5* in the LV wall is transmural whereas expression is limited in the RV. In the forming compact myocardium from E12-13 onwards, *Gja5* is gradually downregulated while it remains expressed in the ventricular trabeculae (Fig. 2b). This indicated that the forming PVCS and ventricular compact myocardium both originate from the *Gja5*^+^ (and *Nppa*^+^) embryonic ventricular wall. It also suggested that the PVCS forms by maintaining the trabecular phenotype, whereas the compact myocardium diverges [44]. Initially, the *Tbx3*^+^ AVB and proximal BBs do not express *Gja5*, but expression is activated gradually after E13-14 of mouse development. Taken together, spatio-temporal gene expression patterns during embryonic ventricular development suggested that the AVB and proximal BBs originate from G1N2^+^ *Tbx3*^+^ *Gja5*^−^ interventricular ring, whereas the distal BBs and the PVCS originate from *Gja5*^+^ G1N2^−^ *Tbx3*^−^ trabeculae.

The first lineage tracing study on the origin of the VCS showed that the PVCS and ventricular working myocardium share a common progenitor pool [46], similar to the suggested origin based on *Gja5* and *Nppa* expression. *In ovo* labeling of avian RV cardiomyocytes by microinjection of a reporter-carrying retrovirus that randomly integrated in the host genome was used to perform a clone analysis. It showed that PVCS and surrounding working myocardium are closely related. The AVB was not labeled in this study [46]. However, the same group did address the origin of the AVB and BBs in a later study. The same method was used and extended by high titer injection of reporter-carrying adenoviruses into the pericardial cavity. Similar to the PVCS, the progenitors of the AVB and BBs contribute to both VCS cells and working myocardium. At the same time, they also showed that VCS cells are more closely related to surrounding working myocardium than with more distant VCS cells [47].

Using current lineage analysis tools, retrospective clonal analysis (low-frequency recombination of *nLaacZ)* and genetic inducible fate mapping (*Gja5-CreER*^*T2*^*-IRESmRFP* and *R26R-YFP* reporter), the common progenitor pool for the VCS and ventricular chambers was confirmed [24]. Moreover, these models allowed for a more thorough investigation of the developmental mode and revealed a progressive restriction towards the VCS followed by limited outgrowth. In detail, double transgenic embryos containing *nLaacZ*, to determine clonal relationships between cells, and *Gja5*^+/GFP^, to discriminate the VCS, were generated. Analysis of the clone cluster size and cluster composition (containing only *Gja5*^+^ conductive cells, only *Gja5*^−^ working myocardium, or both) established the developmental mode of the VCS. From the analysis, it was deduced that the VCS develops by specification to a conductive phenotype (*Gja5*^+^) followed by a limited outgrowth. In parallel, it was shown that the VCS develops by a progressive restriction. Labeling *Gja5*^+^ cardiomyocytes at early developmental stages gave rise to labeled cells within the *Gja5*^+^ VCS and *Gja5*^−^ chamber myocardium (Fig. 2c). Consecutive labeling at later developmental stages revealed larger contributions to *Gja5*^+^ VCS cells and smaller contributions to *Gja5*^−^ working myocardial cells. Complete cell fate restriction of VCS progenitors to the VCS was established around E16.5 in mouse development [24].

It is still not properly established from which cardiogenic mesodermal population the VCS originates. The *Mef2c-AHF-Cre* lineage tracing labels the RV, outflow tract and interventricular septum including the AVB. However, *Cre* remains expressed in the RV, interventricular septum, and AVB at the analysis stage and because of this, labeled cells cannot be traced back to the *Mef2c-AHF*^+^ mesodermal cells [39, 40]. A *Mesp1*^+^ fate map showed that not all AVB and BBs were labeled and concluded that a fraction possibly originates from a *Mesp1*^−^ mesodermal population [48]. This contradicts earlier *Mesp1* lineage tracing that showed all cardiomyocytes in the E9.5 heart to be labeled [49]. Possibly, incomplete labeling of the *Mesp1*^+^ progenitor population plays a role in these contradicting observations. As mentioned in the AVN/AVRBs section, a neural crest (Wnt1-Cre) contribution to cardiomyocytes in the interventricular septum is unlikely either [38, 41].

## Conclusions

A number of studies focused on lineage tracing in the mouse and avian heart have identified the locations of the CCS progenitor cells from the cardiogenic mesoderm to the formed adult heart and showed multiple CCS lineages segregating early in development. The SV/SAN progenitors are located in the most caudal part of the SHF and their fate is specified at that developmental stage and before differentiation towards a cardiomyocyte phenotype occurs. The posterior part of the cardiac crescent is a FHF- and SHF-derived region that forms the IFT of the primary heart tube and subsequently the AVC and finally, in the formed heart, gives rise to the AVN, AVRBs, and part of the LV. The AVB and proximal BB progenitors are added to the arterial pole of the primary heart tube and are therefore part of the embryonic OFT from which later the interventricular septum/ring is formed. The left ventricular PVCS and distal LBB, like the rest of the LV, is a derivative of the FHF that initially formed the primary heart tube. As a consequence, the right ventricular PVCS and distal RBB originates from the SHF. The VCS components and surrounding ventricular cardiomyocytes are closely related and originate from a common progenitor pool. The SV/SAN, AVN/AVRBs, AVB and proximal BBs, left ventricular PVCS, and right ventricular PVCS originate from separate lineages that as a result of cell fate decisions segregate early in embryonic development. This results in sharp boundaries between the CCS components while remaining interconnected to each other (Fig. 1).
